# Controlling frequency dispersion in electromagnetic invisibility cloaks

**DOI:** 10.1038/s41598-019-42481-7

**Published:** 2019-04-15

**Authors:** Geoffroy Klotz, Nicolas Malléjac, Sebastien Guenneau, Stefan Enoch

**Affiliations:** 1 CEA DAM/Le Ripault, BP 16, F-37260 Monts, France; 2 Aix Marseille Univ, CNRS, Centrale Marseille, Institut Fresnel, Marseille, France

## Abstract

Electromagnetic cloaking, as challenging as it may be to the physicist and the engineer has become a topical subject over the past decade. Thanks to the transformations optics (TO) invisibility devices are in sight even though quite drastic limitations remain yet to be lifted. The extreme material properties which are deduced from TO can be achieved in practice using dispersive metamaterials. However, the bandwidth over which a metamaterial cloak is efficient is drastically limited. We design and simulate a spherical cloak which takes into account the dispersive nature of relative permittivity and permeability tensors realized by plasma-like metamaterials. This spherical cloak works over a broad frequency-band even though these materials are of a highly dispersive nature. We establish two equations of state that link the eigenvalues of the permittivity and permeability tensors in every spherical cloak regardless of the geometrical transformation. Frequency dispersive properties do not disrupt cloaking as long as the equations of states are satisfied in the metamaterial cloak.

## Introduction

Transformation optics (TO) is a powerful tool allowing us to imagine complex structured media that control the propagation of electromagnetic waves as if space was distorted. Thirteen years ago, research groupings of Pendry^1^ and Leonhardt^2^ have independently shown how a special distribution of matter in a real physical space can mimic a distorted virtual electromagnetic space. Numerical simulations have validated the TO theory beyond doubt, notably for an electromagnetic source in the far field^3^, and in the intense near field^4^. However, experimental realizations remain challenging: the design of the metamaterial coating must exhibit highly anisotropic effective tensors of relative permittivity and permeability that correspond to the spatially varying tensors given by the TO theory. A non-magnetic cylindrical cloak operating at optical wavelengths has been proposed with a reduced set of parameters, but the cloak’s outer boundary is not perfectly matched with vacuum and thus some reflection persists^5^. In fact, effective tensors of relative permittivity and permeability within the mantle cloak are characterized by eigenvalues ranging from 0 to 1 for a spherical cloak (and from 0 to infinity for a cylindrical cloak), if one wants to realize a mantle in free space. Therefore, we would like to address here three main issues: extreme anisotropy, inhomogeneity and values of relative permittivity and relative permeability bellow unity.

In practice, values of relative permittivity and permeability bellow unity are achievable using resonant metallic inclusions in the coating. Schurig *et al.* achieved the first experimental demonstration in 2006^6^ with concentric rings of split ring resonators. With the use of resonant devices comes one of the challenges of cloaking: the dispersion of materials makes it almost impossible for the coating to operate over a range of frequencies. Many strategies have been tested in order to enlarge the frequency band^7–10^, some of them sacrifice the cloaking efficiency to broaden the bandwidth^11^. Limitations of cloaking with dispersive materials have been further analyzed in^12–14^.

The fundamental concept of TO consists in mimicking a distorted space using a special design of matter. In order to do so we have to define in the first place a mathematical transformation between normal space and the distorted one we want to mimic. We can theoretically choose any one-to-one transformation of our choice, as soon as it is differentiable with respect to the spatial coordinates. By doing so, we can calculate the values of permittivity and permeability tensors required in the cloak. However, these values are function of space coordinates but do not depend on the frequency of the incident wave: in other words, if one wants to create a cloaking device working over a band of frequencies, one has to realize the structure with materials that preserve the targeted electromagnetic properties over the whole frequency band. This is a big issue for values of relative permittivity and permeability bellow unity that we mentioned above.

Kildishev *et al.* published an interesting study ten years ago^15^, about the engineering of dispersion. Considering a first order Taylor approximation for the dispersion of permittivity and permeability, they obtained for a cylindrical cloak with the electric field polarized along the (out-of-plane) z-axis (p-polarization), an elegant formula that exhibits a link between the space transformation and frequency. In other words, authors found that dispersion of material properties can be taken into account and could allow a cloaking over a bandwidth. This approach generates a frequency-dependent mathematical transformation near the initial operating frequency. Besides, Rajput and Srivastava performed simulations of a cylindrical cloak with an extended bandwidth thanks to a similar perturbative approach^16^.

We present here a more general approach of dispersion engineering for spherical cloaks. We propose a convenient and versatile method to design a cloak with dispersive materials.

## Control of Dispersion

Let us consider a transformation that allows a cloaking of a sphere *S*_1_ (of radius *R*_1_) under a sphere *S*_2_ (of radius *R*_2_) with ${R}_{1} < {R}_{2}$. The shell ${S}_{2}-{S}_{1}$ corresponds to the cloaking device. We consider a bijective (one-to-one) and smooth (differentiable) transformation between ${{\mathbb{R}}}^{3}$ and $\{{{\mathbb{R}}}^{3}/{S}_{1}\}$ of the form:

1 $$(r^{\prime} ,\theta ^{\prime} ,\varphi ^{\prime} )=\{\begin{array}{ll}(r,\theta ,\varphi ) & {\rm{if}}\,r > {R}_{2}\\ ({f}^{-1}(r),\theta ,\varphi ) & {\rm{if}}\,r\le {R}_{2}\end{array}$$

TO equations provide us with the formula for the permittivity and permeability tensors. We deduce the tensor ${\overline{\overline{\epsilon }}}_{coating}$ in the spherical coordinates as first established by Dolin in 1961^17^:

2 $$\begin{array}{rcl}{\overline{\overline{\epsilon }}}_{coating}(r)) & = & {(\begin{array}{ccc}\frac{f{(r)}^{2}}{{r}^{2}}\frac{1}{\frac{\partial f(r)}{\partial r}} & 0 & 0\\ 0 & \frac{\partial f(r)}{\partial r} & 0\\ 0 & 0 & \frac{\partial f(r)}{\partial r}\end{array})}_{(r,\theta ,\varphi )}\\  & = & {(\begin{array}{ccc}{\epsilon }_{radial} & 0 & 0\\ 0 & {\epsilon }_{ortho} & 0\\ 0 & 0 & {\epsilon }_{ortho}\end{array})}_{(r,\theta ,\varphi )}\end{array}$$

**Figure 1:**
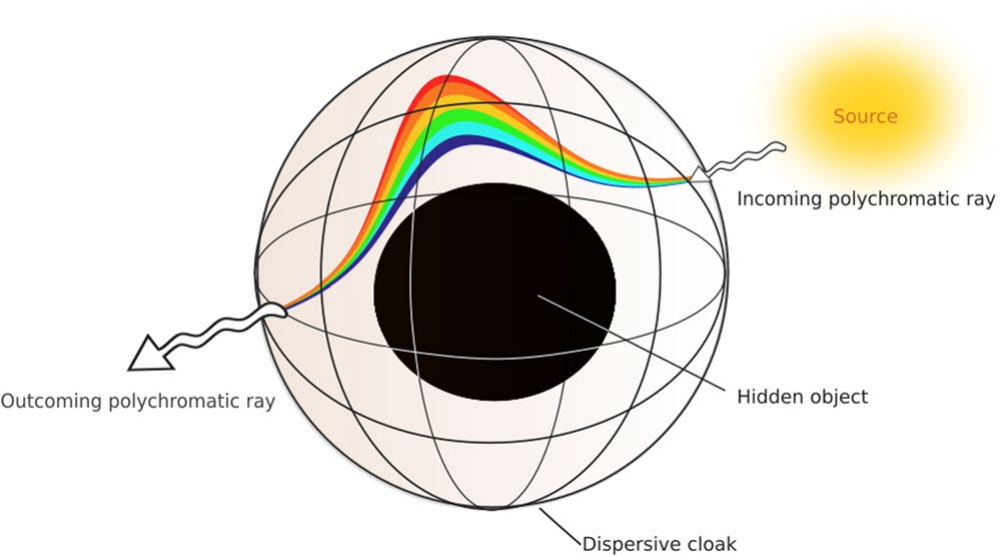
Illustration of rainbow effect within a frequency-dispersive invisibility cloak: inside the cloak, the polychromatic ray is broken up into its spectral constituents. Every color follows its own path so as to avoid the central region. An external observer can neither see the cloak nor the object: he has the illusion that space is empty.

We have seen in the introduction that for a metamaterial cloak, radial permittivity and permeability are necessarily dispersive. We asked ourselves whether it would be possible to engineer a cloaking device that fits the dispersion of materials, by using a frequency dependent geometric transformation. In such a cloak, the path of an incoming wave would depend on its frequency (see Fig. 1).

A simple calculation leads to system (3), in which we consider that the geometric transformation depends on the angular frequency $\omega$:

3 $$\{\begin{array}{c}{\epsilon }_{radial}(r,\omega ){\epsilon }_{ortho}(r,\omega )=\frac{f{(r,\omega )}^{2}}{{r}^{2}}\\ {\epsilon }_{ortho}(r,\omega )=\frac{\partial f(r,\omega )}{\partial r}\end{array}$$

System (3) is written here for the permittivity components, but takes the same form with permeability components. We assume in the sequel that ${\epsilon }_{radial}={\mu }_{radial}$ and ${\epsilon }_{ortho}={\mu }_{ortho}$. As soon as there is a function *f* that allows the one-to-one correspondence (bijection) between ${{\mathbb{R}}}^{3}$ and $\{{{\mathbb{R}}}^{3}/{S}_{1}(\omega )\}$ and such that (3) is fulfilled, cloaking works.

Let us recast (3) to get more convenient criteria that ${\epsilon }_{ortho}$ and ${\epsilon }_{radial}$ have to satisfy in order to achieve cloaking. We note that in (3) equations link ${\epsilon }_{radial}$, ${\epsilon }_{ortho}$, and f: it is thus possible to manipulate equations (see supplemental material) in order to make *f* disappear. We obtain two equivalent systems that are also equivalent to (3) as long as some classical hypotheses on the integrability and derivability of ${\epsilon }_{radial}$ and ${\epsilon }_{ortho}$ (and likewise for the permeability) are verified:

4 $$\{\begin{array}{c}\exists \,{R}_{1}(\omega )/{\int }_{{R}_{1}(\omega )}^{{R}_{2}}\,{\epsilon }_{ortho}(r,\omega )dr={R}_{2}\\ {\epsilon }_{radial}(r,\omega )=\frac{1}{{\epsilon }_{ortho}(r,\omega )}{(\frac{{R}_{2}-{\int }_{r}^{{R}_{2}}{\epsilon }_{ortho}(s,\omega )ds}{r})}^{2}\end{array}$$

5 $$\{\begin{array}{c}\exists \,{R}_{1}(\omega )/{li}{{m}}_{r\to {R}_{1}(\omega )}({\int }_{r}^{{R}_{2}}\,\frac{dx}{{x}^{2}{\epsilon }_{radial}(x,\omega )})=+\,\infty \\ {\epsilon }_{ortho}(r,\omega )=\frac{1}{{\epsilon }_{radial}(r,\omega ){r}^{2}{(\frac{1}{{R}_{2}}+{\int }_{{R}_{2}}^{r}\frac{-1}{{s}^{2}{\epsilon }_{radial}(s,\omega )}ds)}^{2}}\end{array}$$

In (4) and (5), the geometric transformation is implicit. We have here two equations in each system:

- First equation of each system gives a criterion on ${\epsilon }_{ortho}$ or ${\epsilon }_{radial}$ that has to be verified if we want a bijection between ${{\mathbb{R}}}^{3}$ and $\{{{\mathbb{R}}}^{3}/{S}_{1}(\omega )\}$
- Second equation of each system gives a constitutive relation between the components of the permittivity (and also permeability) tensor. This ensures the required optical path, in the light ray point of view, in the coating.

The main idea that emerges from these equations is that as soon as radial components of permittivity and permeability are small enough, there is a function that gives the orthordial permittivity and permeability in order to realize a cloak. Conversely, as soon as we have large enough orthoradial components, there exists a function that describes the radial components.

## Application

We now consider as an illustrative example a cloak for which the radial components of permittivity and permeability behave as an ideal lossless Drude metal near its plasma frequency. Let us set the plasma frequency as a function of radius, ${\omega }_{p}=\frac{{R}_{2}-r}{{R}_{2}-{R}_{1}}{\omega }_{0}$, then we deduce the radial permittivity and permeability:

6 $${\epsilon }_{radial}(r,\omega )={\mu }_{radial}(r,\omega )=1-\frac{{\omega }_{0}^{2}}{{\omega }^{2}}{(\frac{{R}_{2}-r}{{R}_{2}-{R}_{1}})}^{2}$$

Frequency ${\omega }_{0}$ is the maximum frequency of operation of the cloak. The components of the permittivity and permeability tensors are known, so it is possible to perform numerical simulations. In order to simulate a frequency dependent cloaking, we chose to design a multilayered cloak: each layer of the cloak is composed of an anisotropic theoretical material whose permittivity and permeability tensors are diagonal in the spherical coordinates. The choice of a multilayer design limits the number of different plasma frequencies in the cloak, and makes it more realistic: instead of using function (6) as written here, the variable “r” has been discretized, so that tensor components in each layer are constant. We created 20 materials with the COMSOL interface, whose permittivity and permeability tensors are calculated as follow. The radial components of permeability and permittivity have a plasma-like dispersive behavior and the orthoradial properties are deduced using the constitutive equation of system (5). To obtain constant tensor components in the spherical coordinates we assume that the radius is constant inside each layer: the thickness of each layer is supposed to be small compared to its radius of curvature. The plasma frequency is then given by:

7 $${\omega }_{p,layer}={\omega }_{p}({r}_{layer})=\frac{{R}_{2}-{r}_{layer}}{{R}_{2}-{R}_{1}}{\omega }_{0}$$

We deduce the permittivity and permeability tensors:

8 $${\overline{\overline{\epsilon }}}_{layer}(\omega )={\overline{\overline{\mu }}}_{layer}(\omega )=(1-\frac{{\omega }_{p,layer}^{2}}{{\omega }^{2}}){(\begin{array}{ccc}1 & 0 & 0\\ 0 & {\alpha }_{layer}(\omega ) & 0\\ 0 & 0 & {\alpha }_{layer}(\omega )\end{array})}_{(r,\theta ,\varphi )}$$

with *α*_*layer*_ a function of the frequency that describes the anisotropy of the material:

9 $${\alpha }_{layer}(\omega )={(1-\frac{{\omega }_{p,layer}^{2}}{{\omega }^{2}})}^{-2}{(\frac{{r}_{layer}}{{R}_{2}}+{\int }_{{R}_{2}}^{{r}_{layer}}\frac{-{r}_{layer}}{{s}^{2}(1-\frac{{\omega }_{p}{(s)}^{2}}{{\omega }^{2}})}ds)}^{-2}$$

**Figure 2:**
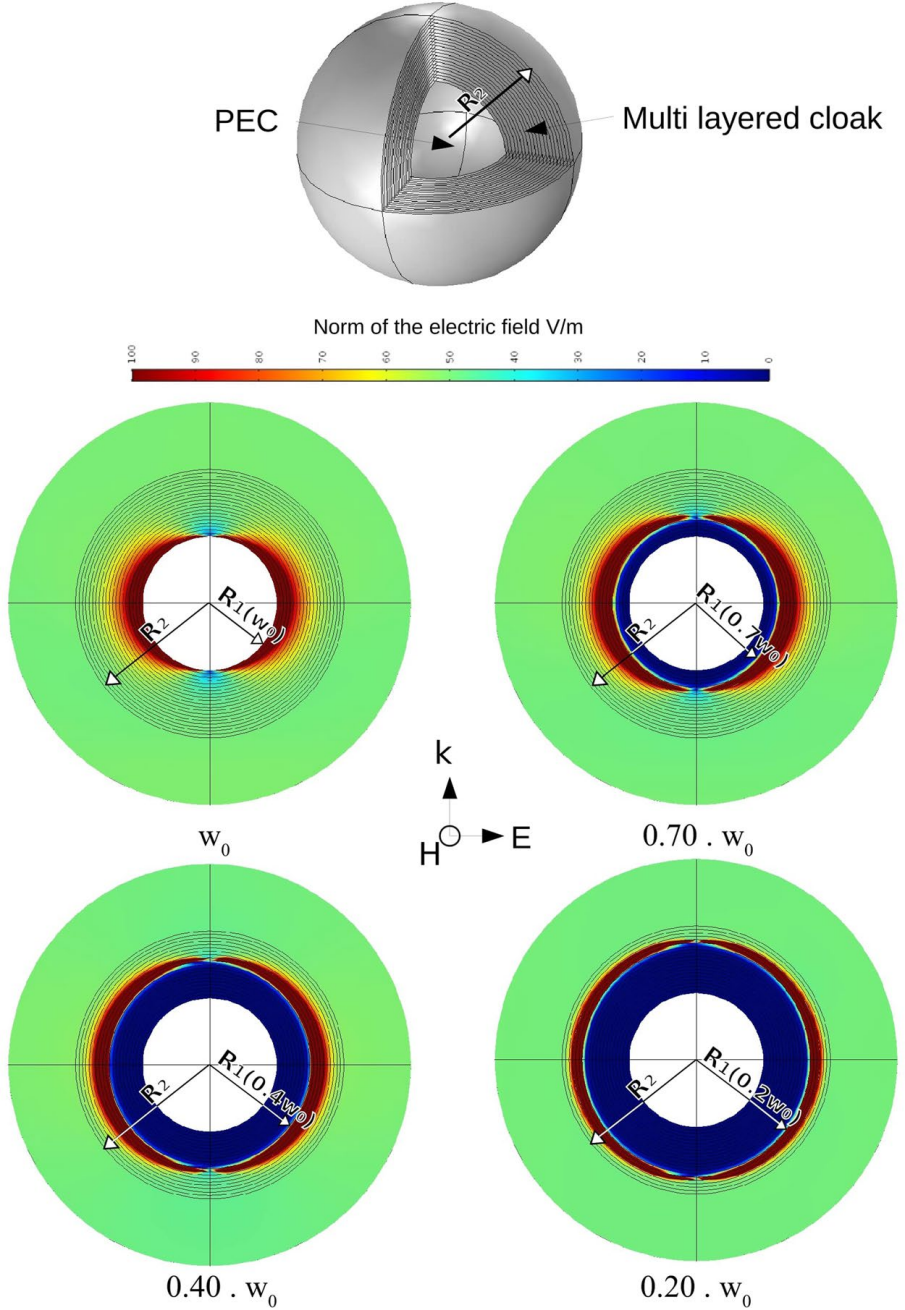
Norm of the electric field for different frequencies: simulation of the dispersion engineered cloak. The magnetic field of the source is oriented perpendicularly to the cut plane. For these simulations, ${R}_{1}({\omega }_{0})=0.5{R}_{2}$, ${\lambda }_{0}=3{R}_{2}$ with *λ*_0_ the wavelength in the vacuum at the frequency ${\omega }_{0}$. The cloaked region can be visually identified because no electric field can enter in: this is the blue region growing up around the perfect electric conductor as the frequency decreases.

**Figure 3:**
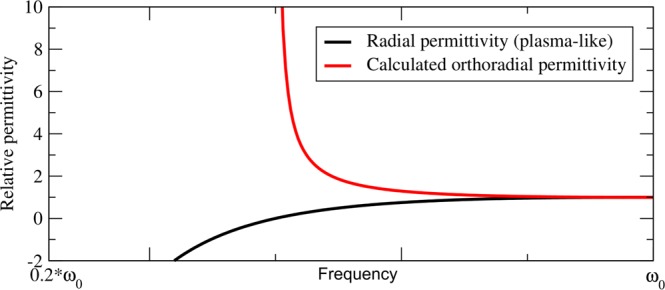
Typical radial and orthoradial permittivity and permeability of a material composing one of the layers in the cloak. The black curve gives de dispersive behaviour of the plasma-like radial permittivity, whereas the red curve gives the orthoradial permittivity that was calculated using the second equation of (5). The plasma frequency is ${\omega }_{p}={\omega }_{0}$/2, and it corresponds to the material in the 10^th^ layer (counting from the outside of the cloak to the inside).

Full wave simulations have been performed with *COMOL Multiphysics*^®^ and validate the theory in the frequency domain: results can be seen on Fig. 2 and in supplemental material. Every layer of that cloak is composed of an adapted theoretical material as previously described. We assigned to each layer its own material and we assigned normal air (equivalent to free space) to the region outside the cloak. Radial and orthoradial properties of a representative layer can be seen on Fig. 3.

The sphere of radius ${R}_{1}({\omega }_{0})$ inside the coating is a perfect conductor that exhibits a large Radar Cross Section in the absence of cloaking. The maximum element size of the mesh we used for the calculation is equal to *λ*_0_/10, with *λ*_0_ the wavelength in free space for the frequency ${\omega }_{0}$. We used the scattered field module of COMSOL, with a background linear polarized electromagnetic wave: its electric field is oriented horizontally, and its magnetic field is oriented perpendicularly to the cut plane.

The wave vector is oriented vertically on Fig. 2 so that we can note that the cloak is working well. Indeed, we neither observe a shadow forward nor wave interference backward. The blue region we can observe on Fig. 2, where no electric field can enter in, is the cloaked region: its size depends on the frequency of the background wave. Indeed, radius ${R}_{1}(\omega )$, that is the radius of the cloaked region at frequency $\omega$, is growing up as the frequency decreases. In the red regions the electric field is stronger: it correspond to the regions where the radial permittivity reaches values near zero.

**Figure 4:**
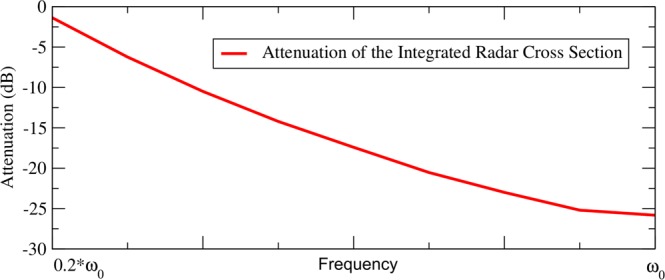
Attenuation (dB) of the Integrated Radar Cross Section of the cloaked metallic sphere. The closer we are to ${\omega }_{0}$, the more layers contribute to the cloaking: this explains why we have a better efficiency near ${\omega }_{0}$. These results have been obtained with a total of 20 layers, and could be improved if we consider more layers.

We can observe on Fig. 2 that the cloak is working over a frequency band from 0.2 ${\omega }_{0}$ to ${\omega }_{0}$. Over that interval of frequencies the norm of the scattered far field remains very low in comparison with the metallic sphere without the cloak (see Fig. 4). The maximal theoretical band of operation is contained in the interval  $[{\omega }_{{\min }},{\omega }_{0}]$ where ${\omega }_{{\min }}$ is given by the plasma frequency at the external boundary of the cloak: ${\omega }_{{\min }}=0$ theoretically at $r={R}_{2}$. In other words, we can cloak with this design over the entire frequency band within which we are able to obtain a plasma-like resonance.

As in^15^ and^16^ we obtain an internal radius that varies with the frequency. Looking at the simulations, that phenomenon can be seen very clearly. We can also clearly identify that there is no electric field near the metallic sphere, inside the frequency-dependent cloaked region. The reduction of Radar Cross Section between the metallic sphere uncloaked and cloaked, reaches typical values for cloaking using a multilayered cloak. The attenuation of the Radar Cross Sections, which is proportional to the square of the norm of the scattered far field is represented in Fig. 4. The reduced attenuation for low frequencies is caused by the reduction of the number of layers that contribute to the cloaking. Indeed, ${R}_{1}(\omega )$ becomes larger for low frequencies (see Fig. 2), so that the invisible region includes some of the layers as soon as we illuminate with frequencies below ${\omega }_{0}$. For a given frequency $\omega  < {\omega }_{0}$, layers that are included in the sphere of radius ${R}_{1}(\omega )$ do not contribute to the cloaking. The reduction of contributing layers at low frequencies has a negative impact on its efficiency.

The attenuation can theoretically be infinite for continuously varying materials as soon as and the components of permeability and permittivity tensors satisfy the equations over the frequency band of interest. Indeed, no approximation has been done to derive the equations.

One of the limitations of our method is the exotic dispersion profile that we obtain for ${\epsilon }_{ortho}$. We cannot guarantee that a material exhibiting such properties exist. One possibility is to design a metamaterial consisting of highly conducting rods and wires, as homogenization theory ensures us that eigenvalues of effective permittivity and permeabilty can approximate any real values in the microwave regime^18^. Another possibility to achieve the strange properties showed in Fig. 3 could possibly be approximated with a combination of a high pass filter or even band pass filters and regular dielectrics. In the field of microwave frequencies, one can use for example high pass filters realized with microstrip lines imitating a T-circuit^19^, whereas band pass filters are very satisfying using rectangular complementary split ring resonators^20^. A thorough investigation of combinations of dispersive materials in the metamaterial of the coating could lead to a good approximation of the desired permittivity and permeability dispersion.

**Figure 5:**
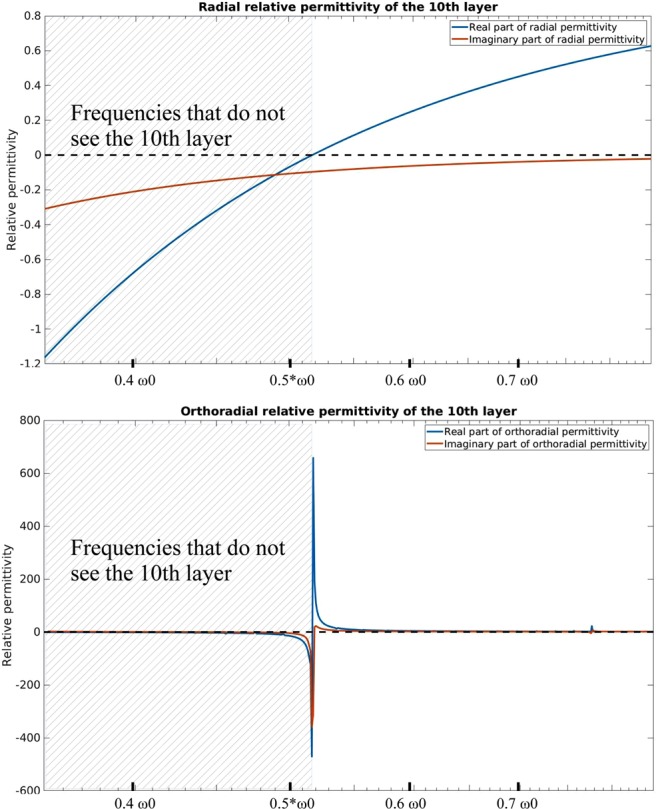
Real and imaginary part of the radial and orthoradial permittivity. Radial permittivity behaves as a Drude metal, whereas the real part of the orthoradial permittivity is deduced using our methodology. Then, the imaginary part of the orthoradial permittivity is calculated using the Kramers-Krönig relations.

Our illustrative example remains highly theoretical, because suitable metamaterials, exhibiting radial and orthoradial properties of Fig. 3 need be designed for instance using homogenization or retrieval techniques. In our example losses are neglected, that basically contradicts the Kramers-Krönig relations and causality. Indeed, we decided to focus on propagating modes to introduce the concept of the frequency-dispersive cloak. In Fig. 5 we give an estimation of the imaginary parts of the radial and orthoradial permittivity. Radial permittivity follows the Drude-model, whereas the orthoradial permittivity has an exotic dispersion: to obtain its imaginary part, we used the Kramers-Krönig relations. For the Drude model, we assumed ${\omega }_{p}\tau =10$.

10 $$\{\begin{array}{c}{Im}({\epsilon }_{radial}(\omega ))=-\,\frac{{\omega }_{p}^{2}{\tau }^{2}}{\omega \tau (1+{\omega }^{2}{\tau }^{2})}\\ {Im}({\epsilon }_{ortho}(\omega ))=\frac{-2\omega }{\pi }\,{\int }_{0}^{\infty }\,\frac{{Re}({\epsilon }_{ortho}(\omega ^{\prime} ))-1}{{\omega ^{\prime} }^{2}-{\omega }^{2}}d\omega ^{\prime} \end{array}$$

Further work could be done to measure the impact of losses on the efficiency of the frequency-dispersive cloak, as it has been done for regular cloaks^21,22^. As a consequence of causality, an electromagnetic pulse composed of a whole frequency spectrum passing through the cloak would necessarily be delayed and spread over time. Our theory and simulations are thereby relevant only in the frequency domain with temporally extended periodic signals, and do not require a violation of causality. Indeed, as soon as the phase and path of the incident waves are correct after travelling through the cloak, an observer cannot distinguish any difference between this signal and the wave that would have travelled through free space.

## Conclusion

Transformation optics allows us to imagine many ways to cloak objects as soon as we are able to manufacture the coating. Nonetheless, one of the main limitations to cloaking is the bandwidth, that cannot easily be enlarged. The system of equations we set up allows us to design broadband cloaking using dispersive materials. We cannot force materials to be non-dispersive, and moreover we cannot impose a specific dependence between permittivity (or permeability) and frequency. However, thanks to existing materials and with metamaterials technology at hand, we believe it may be possible to design a broadband cloak by following the proposed strategy.

## Supplementary information

Supplemental: Controlling frequency dispersion in electromagnetic invisibility cloaks
