# Supplementary material for: Controlling frequency dispersion in electromagnetic invisibility cloaks
Source: Sci Rep. 2019 Apr 15;9:6022. doi: 10.1038/s41598-019-42481-7 (PMC6465290; doi:10.1038/s41598-019-42481-7)
Supplement: Supplementary file 1 — Supplemental: Controlling frequency dispersion in electromagnetic invisibility cloaks [file 41598_2019_42481_MOESM1_ESM.pdf]

Supplemental: Controlling frequency dispersion in electromagnetic invisibility cloaks

Geoffroy Klotz[1,2], Nicolas Malléjac[1] , Sebastien Guenneau[2], Stefan Enoch[2]

March 18, 2019

1 : CEA DAM/Le Ripault, BP 16, F-37260 Monts, France
2 : Aix Marseille Univ, CNRS, Centrale Marseille, Institut Fresnel, Marseille, France

1 Derivation of frequency-dispersive cloak's parameters

Let us consider a transformation that allows for a cloaking of a sphere S_1 (of radius R_1) under a sphere S_2 (of radius R_2) with $R_1 < R_2$. The shell $S_2 - S_1$ corresponds to the cloaking device. We consider a bijective and smooth transformation between $\mathbb{R}^3$ and $\{\mathbb{R}^3/S_1\}$ of the form :

$$(r', \theta', \phi') = \begin{cases} (r, \theta, \phi) & if\ r > R_2 \\ (f^{-1}(r), \theta, \phi) & if\ r \leq R_2 \end{cases} \tag{1}$$

Tools of transformation optics (TO) lead to permittivity and permability tensors as functions of the radius r :

$$\bar{\bar{\epsilon}}_{coating}(r) = \bar{\bar{J}}^{-1}(r)\ \bar{\bar{\epsilon}}_{medium}(r)\ \bar{\bar{J}}^{-T}(r)\ det(\bar{\bar{J}}(r)) \tag{2}$$

If the background medium is air we have $\bar{\bar{\epsilon}}_{medium} = I_3$, the identity matrix. J stands for the jacobian matrix of the transformation f. We deduce the tensor $\bar{\bar{\epsilon}}_{coating}$ in the spherical coordinates as established by L.S. Dolin in a paper that predates TO : "On the possibility of comparison of three-dimensional electromagnetic systems with nonuniform anisotropic filling" :

$$\bar{\bar{\epsilon}}_{coating}(r)) = \bar{\bar{\mu}}_{coating}(r)) = \begin{pmatrix} \frac{f(r)^2}{r^2} \frac{1}{\frac{\partial f(r)}{\partial r}} & 0 & 0 \\ 0 & \frac{\partial f(r)}{\partial r} & 0 \\ 0 & 0 & \frac{\partial f(r)}{\partial r} \end{pmatrix}_{(r,\theta,\phi)} \tag{3}$$

We deduce the following system :

$$\begin{cases} \epsilon_{radial}(r)\epsilon_{ortho}(r) = \frac{f(r)^2}{r^2} \\ \epsilon_{ortho}(r) = \frac{\partial f(r)}{\partial r} \end{cases} \tag{4}$$

We then consider that the whole system is frequency-dependent, in order to take into account the dispersion of materials :

$$\begin{cases} \epsilon_{radial}(r,\omega)\epsilon_{ortho}(r,\omega) = \frac{f(r,\omega)^2}{r^2} \\ \epsilon_{ortho}(r,\omega) = \frac{\partial f(r,\omega)}{\partial r} \end{cases} \tag{5}$$

For given permeability components, as soon as there is a function f that allows the bijection between $\mathbb{R}^3$ and $\{\mathbb{R}^3/S_1\}$ and that verifies (5) cloaking is achieved. However such a system is difficult to manipulate : if the radial component of permittivity is imposed by materials and happens to be dispersive, we won't easily find a suitable function f.

We thus transform that system to obtain more convenient criteria that ϵ_{ortho} and ϵ_{radial} have to satisfy in order to achieve cloaking. We can first integrate the second equation between R_1 and R_2, to obtain a criterion on ϵ_{ortho} :

$$\int_{R_1(\omega)}^{R_2} \epsilon_{ortho}(r,\omega)dr = f(R_2) - f(R_1(\omega)))$$

The continuity of the function f gives us $f(R_2) = R_2$ and $f(R_1(\omega)) = 0$. We obtain the following criterion :

$$\exists\ R_1(\omega)\ /\ \int_{R_1(\omega)}^{R_2} \epsilon_{ortho}(r,\omega)dr = R_2 \tag{6}$$

Conversely, considering a given function of radius ϵ_{ortho} , if there exists R_1 for which the integral between R_1 and R_2 of ϵ_{ortho} is equal to R_2 and ϵ_{ortho} is positive and integrable over that interval,then there is a function f for which ϵ_{ortho} is well suited. In that case the function f is the primitive of ϵ_{ortho} that vanishes in R_1 . We can find a similar criterion for ϵ_{radial} that is the counterpart of (6) :

$$\exists\ R_1(\omega)\ /\ lim_{r\to R_1(\omega)} \left(\int_r^{R_2} \frac{dx}{x^2 \epsilon_{radial}(x,\omega)} \right) \to +\infty \tag{7}$$

As soon as ϵ_{ortho} is a function of radius that is positive and integrable, equation (6) and the second equation of the system (5) are equivalent. We will now consider the following system as being equivalent to (5) :

$$\left\{ \begin{array}{l} \epsilon_{radial}(r,\omega)\epsilon_{ortho}(r,\omega) = \dfrac{f(r,\omega)^2}{r^2} \\ \exists\ R_1(\omega)\ /\ \displaystyle\int_{R_1(\omega)}^{R_2} \epsilon_{ortho}(r,\omega)dr = R_2 \end{array} \right. \tag{8}$$

Using the boundary conditions $f(R_2) = R_2$ and $f(R_1) = 0$, we can extract an equation that links ϵ_{radial} and ϵ_{ortho} without an explicit dependence upon f :

$$\epsilon_{radial}(r,\omega) = \frac{1}{\epsilon_{ortho}(r,\omega)} \left(\frac{R_2 - \displaystyle\int_r^{R_2} \epsilon_{ortho}(s,\omega)ds}{r} \right)^2 \tag{9}$$

Equation (9) provides us with the criterion that links the components of permittivity (and also permeability) tensor without explicit dependence upon the geometric transformation. However it would be more convenient to have an equation that gives ϵ_{ortho} as a function of ϵ_{radial} . For this purpose, let us introduce the function α, defined as :

$$\alpha(r,\omega) = \sqrt{\epsilon_{radial}(r,\omega)\epsilon_{ortho}(r,\omega)} \tag{10}$$

When we differentiate the first equation in (8) we obtain :

$$\frac{\partial f(r,\omega)}{\partial r} = \frac{\partial \sqrt{r^2 \epsilon_{radial}(r,\omega)\epsilon_{ortho}(r,\omega)}}{\partial r}$$

$$\epsilon_{ortho}(r,\omega) = \frac{\partial \left(r\alpha(r,\omega) \right)}{\partial r}$$

$$\frac{\alpha(r,\omega)^2}{\epsilon_{radial}(r,\omega)} = \frac{\partial\left(r\alpha(r,\omega)\right)}{\partial r}$$

Calculations lead us then to the following differential equation for α :

$$\frac{\partial\alpha(r,\omega)}{\partial r} + \frac{\alpha(r,\omega)}{r} = \frac{\alpha(r,\omega)^2}{\epsilon_{radial}(r,\omega)r} \tag{11}$$

We recognize a Bernouilli differential equation that can be solved with the boundary conditions we have on α :

$$\alpha(r,\omega) = \frac{1}{r\left(\frac{1}{R_2} + \int_{R_2}^{r} \frac{-1}{s^2\epsilon_{radial}(s,\omega)} ds\right)} \tag{12}$$

We finally obtain ϵ_{ortho} as a function of ϵ_{radial} :

$$\epsilon_{ortho}(r,\omega) = \frac{1}{\epsilon_{radial}(r,\omega)r^2\left(\frac{1}{R_2} + \int_{R_2}^{r} \frac{-1}{s^2\epsilon_{radial}(s,\omega)} ds\right)^2} \tag{13}$$

2 Simulations

Simulations have been performed with the RF module of *COMSOL Multiphysics*® 5.3 in the frequency domain. We created a sphere composed of a perfect electric conductor (PEC), that constitutes the scatterer we want to hide. We created then 20 other concentric spheres, delimiting the 20 different layers composing the cloak. Two additional concentric spheres, larger than the previous ones, delimit the heterogeneous anisotropic and absorptive perfectly matched layer (spherical PML), outside the cloak. This PML is represented on figures 2 and 3.
Each layer is composed of a material whose electromagnetic properties (permittivity and permeability) are anisotropic. We considered a finite number of layers and a discretized radial coordinate in order to have a finite number of materials in the cloak. Of course, these 20 materials are still theoretical and cannot be found in nature : some further work is required to experimentally obtain such a collection of materials. Nonetheless, homogenization theory tells us that for instance a combination of conducting wires and split rings could achieve just these (Milton, "Realizability of metamaterials with prescribed electric permittivity and magnetic permeability tensors"). In a spherical cloak, eigenvectors of permittivity and permeability tensors are collinear to the vectors of the spherical basis. We calculated the eigenvalues of these tensors in the spherical basis and used the Jacobian matrix to convert it into the cartesian basis, that COMSOL understands.
The permittivity and permeability tensors in the layers are frequency-dependent, in order to match those calculated by our methodology : they behave as a plasma in the radial direction, and as a particular sort of high-pass filter in the orthoradial directions.

The mesh was composed of 315 000 tetrahedra (see Fig.2) and was built to make possible a simulation at the highest frequency, that is the frequency of reference of the cloak : we used the same mesh for every frequency. In the same way, the PML thickness was dimensioned in order to work at ω_0.
Using the RF module of COMSOL, we performed our simulations in the frequency domain. The electromagnetic excitation is a background plane wave that is linearly polarized. For the simulations presented in the article, the frequency of the background plane wave is a parameter that takes successively 9 different values between $0.2\omega_0$ and ω_0.
The PML may have caused some slight problems at lower frequency, because it was too thin compared to the wavelength in free space. However, it was difficult to enlarge it, because the number of elements

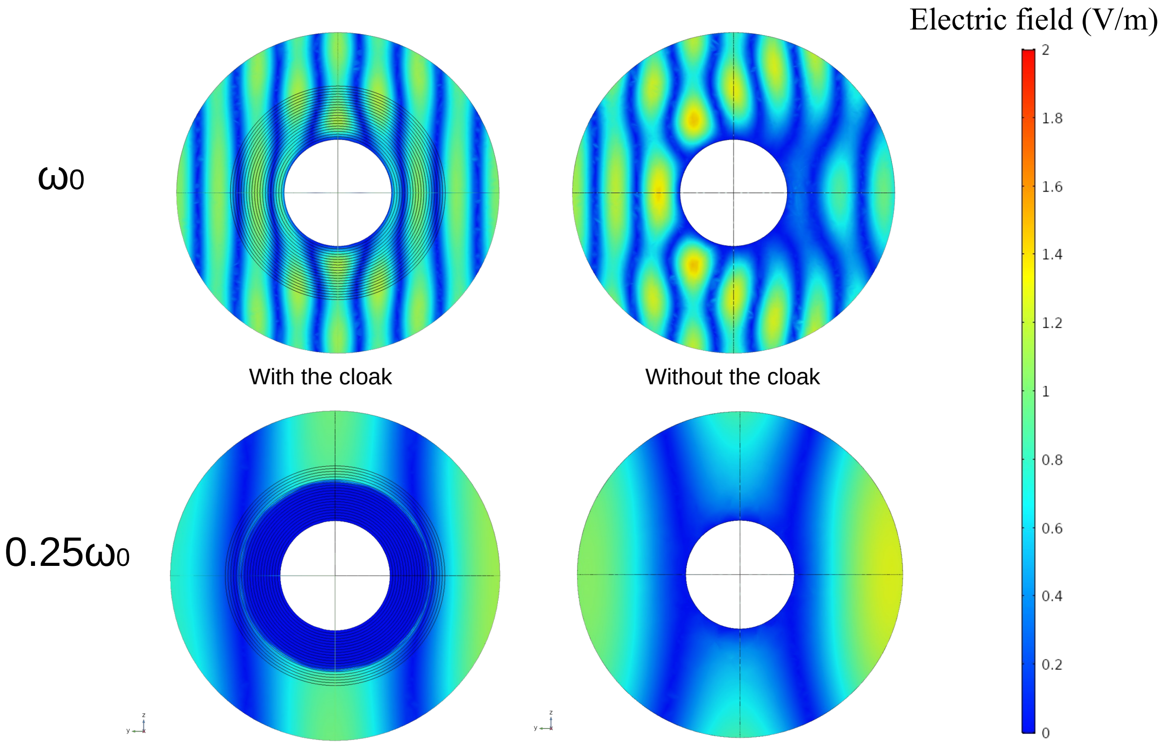

Figure 1: Instantaneous absolute value of the electric field, with and without the cloak around the PEC. Simulation with $\omega_0 = 0.75R_2$, different than the one in the main paper. We can see that the cloaking device allows the propagation of the plane wave, with no apparent perturbation. The incident wave travels from the left to the right, and its electric field is oriented perpendicularly to the plane of the figure.

would have been too large for computational resources available. With the mesh of Fig.2, composed of 315 000 elements of domain, the simulation on a 8-Core processor (Xeon(R) CPU E5-2637 v2) lasted 4 hours for 9 different frequencies : 27 minutes per frequency on average.

3 Computation of the attenuation

In order to evaluate quantitatively the efficiency of our cloaking device in the simulations, we set up the following procedure :

- we perform the numerical simulation of the metallic sphere cloaked with a frequency sweep : the frequency ration ω/ω_0 starts at 0.2 and stop at 1 with a step of 0.1.
- we perform the same simulations without the cloak.
- we extract the norm of the electrical scattered far field along 1250 different directions for each frequency, with and without the cloak.
- we calculate the average far field for each frequency.
- we calculate for each frequency the following attenuation, that is equivalent to the attenuation of the Integrated Radar Cross Section : $A = 10\log\left(\frac{<E_{far,cloaked}>^2}{<E_{far,uncloaked}>^2}\right)$

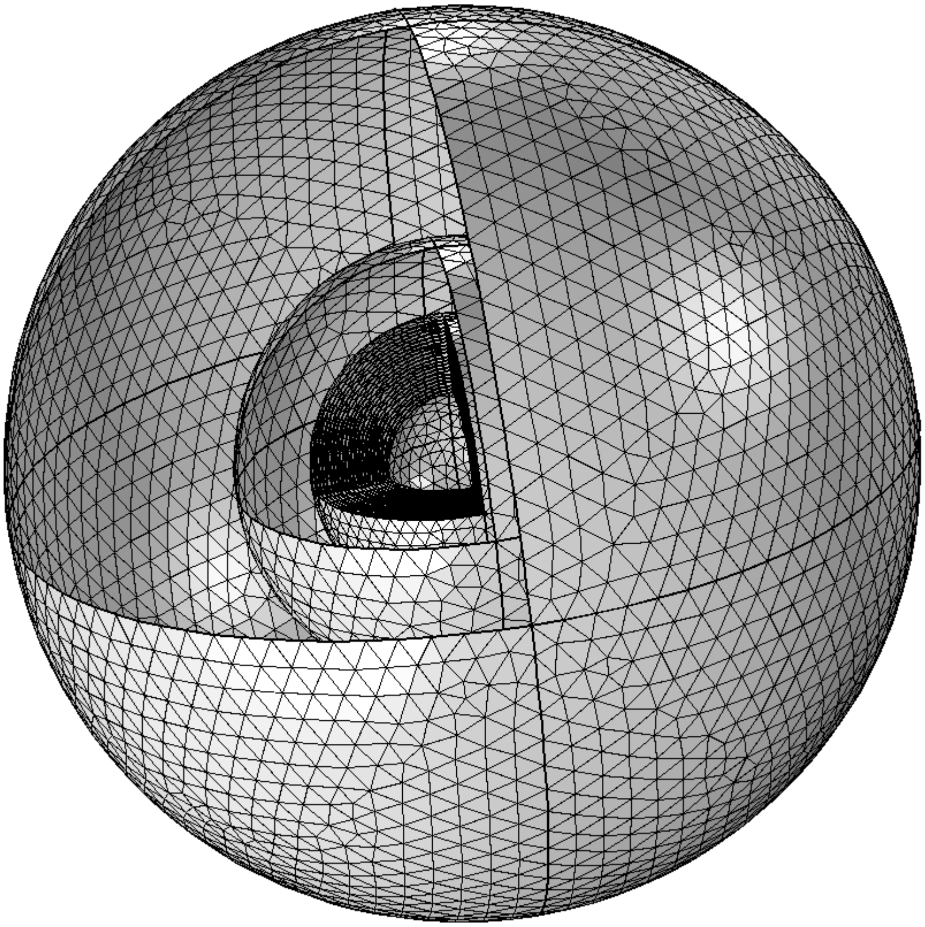

Figure 2: Mesh used for the simulations : the external large layer is the PML region. The maximum size of a mesh element is equal to $\lambda_0/10$, with λ_0 the wavelength in free space for the highest frequency. It is composed of 315 000 domain elements.

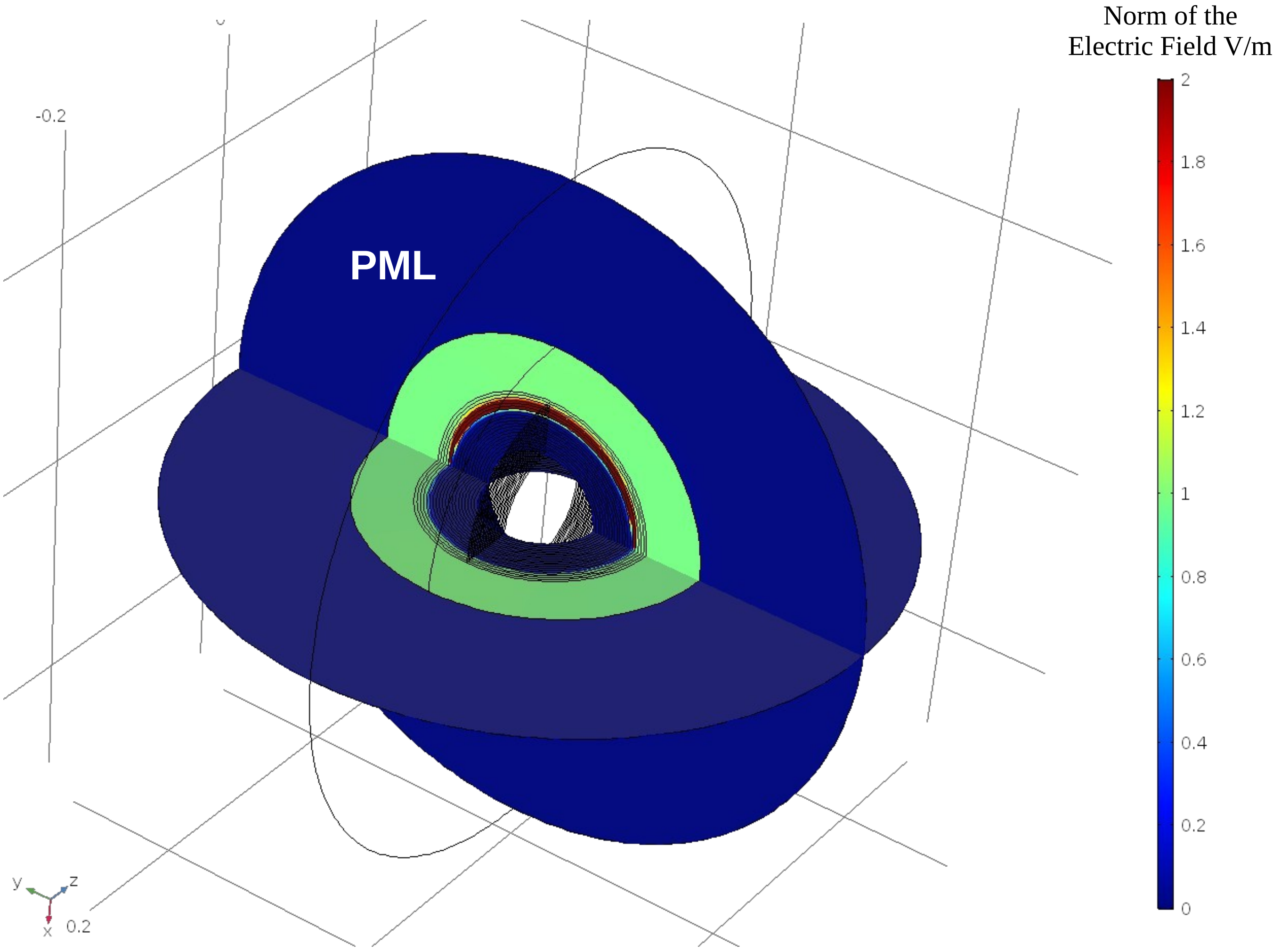

Figure 3: Norm of the electric field for a simulation with $\omega/\omega_0 = 0.2$. The wave vector of the background wave is along the y direction, and the electric field of the background wave is along the x direction.
